# Behavioral Feeding Circuit: Dietary Fat-Induced Effects of Inflammatory Mediators in the Hypothalamus

**DOI:** 10.3389/fendo.2020.591559

**Published:** 2020-11-26

**Authors:** Kinning Poon

**Affiliations:** Department of Biological Sciences, SUNY College at Old Westbury, Old Westbury, NY, United States

**Keywords:** high-fat diet, hypothalamus, maternal high-fat diet, chemokines, orexigenic neuropeptides, inflammation

## Abstract

Excessive dietary fat intake has extensive impacts on several physiological systems and can lead to metabolic and nonmetabolic disease. In animal models of ingestion, exposure to a high fat diet during pregnancy predisposes offspring to increase intake of dietary fat and causes increase in weight gain that can lead to obesity, and without intervention, these physiological and behavioral consequences can persist for several generations. The hypothalamus is a region of the brain that responds to physiological hunger and fullness and contains orexigenic neuropeptide systems that have long been associated with dietary fat intake. The past fifteen years of research show that prenatal exposure to a high fat diet increases neurogenesis of these neuropeptide systems in offspring brain and are correlated to behavioral changes that induce a pro-consummatory and obesogenic phenotype. Current research has uncovered several potential molecular mechanisms by which excessive dietary fat alters the hypothalamus and involve dietary fatty acids, the immune system, gut microbiota, and transcriptional and epigenetic changes. This review will examine the current knowledge of dietary fat-associated changes in the hypothalamus and the potential pathways involved in modifying the development of orexigenic peptide neurons that lead to changes in ingestive behavior, with a special emphasis on inflammation by chemokines.

## Introduction

The latest National Health and Nutrition Examination Survey reports 39.8% of adults and 18.5% of youth in the United States to be obese (1), with the ingestion of a diet rich in saturated fats or high in omega-6 fatty acids to be a risk factor in developing obesity (2–5). The ingestion of a High-fat diet (HFD) in humans and in adult animal models have harmful effects to physiological organ systems by inducing a state of systemic inflammation that is related to the increased risk for developing disease including cardiovascular disease, diabetes, and cancer (6–8), and also has cognitive effects in humans (9, 10). These disease states have been correlated to HFD-induced cellular changes in all organ systems, such as adipose tissue, liver, heart, kidneys, and the gut itself (11, 12). The effects of a HFD are not exhaustive to peripheral organ systems and can also directly impact the central nervous system (13, 14), with the central effects governing ingestive behavior. Ingestion of a HFD and obesity prior to mating, during pregnancy, and postnatally also induces similar physiological and central phenotypic outcome in offspring. Animal studies have revealed a transgenerational effect of HFD intake or obesity from both paternal and maternal sides prior to and during pregnancy on over-consummatory behavior in offspring (15, 16), with similar findings in humans from the 1958 British birth cohort study (17). Recently, the fields of developmental biology, metabolomics, nutrition, and ingestive behavior have linked the systemic and central nervous system effects of a fat-rich diet to the activation of the immune system, with inflammatory mediators playing a potential role in mediating HFD changes on the periphery, the brain and on developmental processes (18, 19). The connectivity of these disciplines on the outcome of HFD intake and obesity bring to light the complexity between inflammation and a HFD in adult animal models and in offspring that are prenatally-exposed to this diet, particularly on specific inflammatory mediators such as the chemokines. This article will begin with a brief historical perspective on ingestive behavior and examine in more detail the intersection of other fields in relation to inflammation by chemokines to uncover cellular mechanisms of HFD effects.

## Dietary Fat on Stimulating Neurochemical Systems in the Hypothalamus

**Figure 1:**
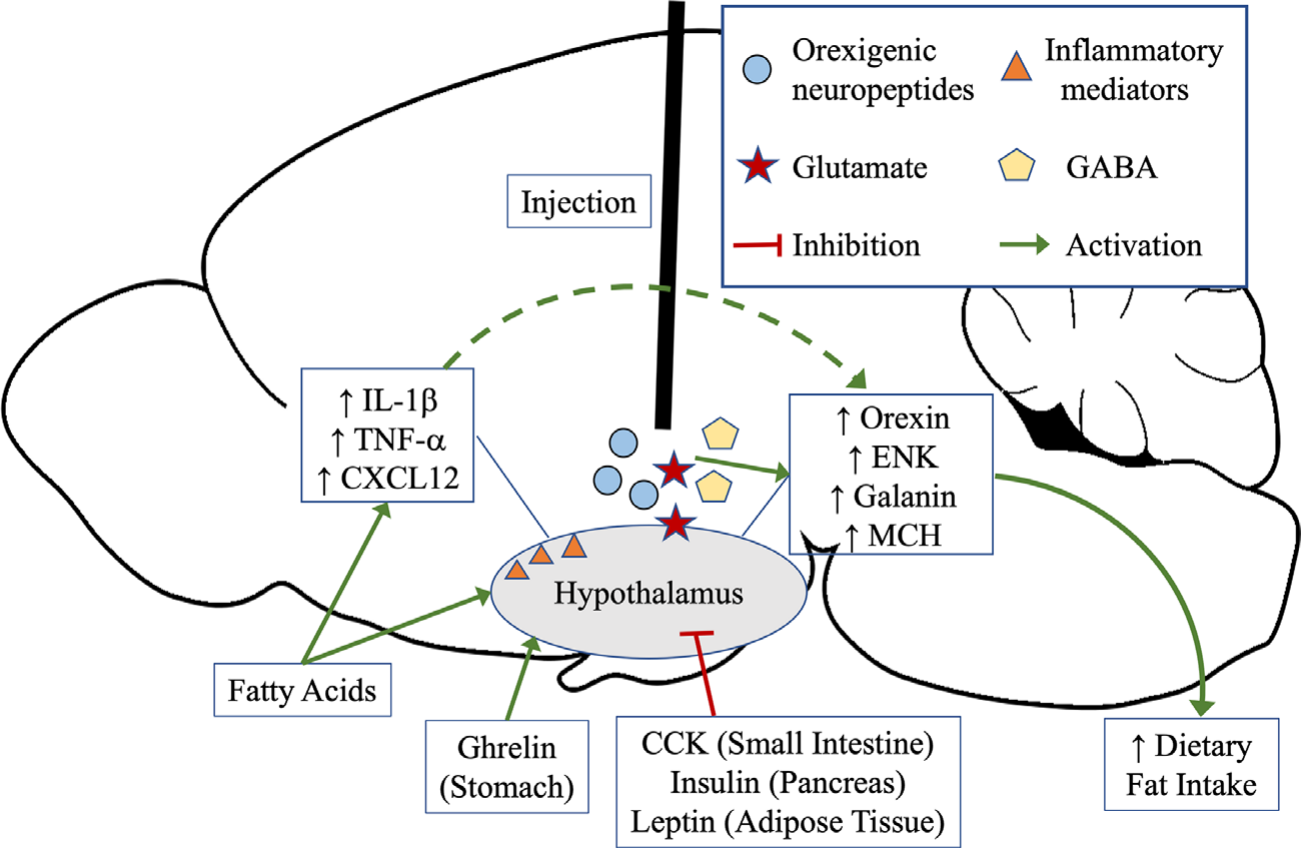
Hunger and satiety can stimulate the release of gut neuropeptides that directly signal the hypothalamus. Ghrelin, released from the stomach during hunger, activates the orexigenic peptide neurons in the hypothalamus to signal food intake, whereas cholecystokinin (CCK) from the intestine, insulin from the pancreas, or leptin from adipose tissue, inhibits orexigenic neuropeptide signaling in the hypothalamus to signal satiety and high levels of macronutrients. Diets rich in fats can directly impact activation of the orexigenic neuropeptide signaling in the hypothalamus, leading to increased ingestive behavior, and additionally induce a state of inflammation. Inflammatory mediators have also been shown to affect orexigenic neuropeptides in the hypothalamus. Other neurotransmitters, such as glutamate and GABA, have also been shown to stimulate orexigenic neuropeptide release and can lead to increase in dietary fat intake.

Several decades of research have well-established in adult animal models that the ingestion of a fat-rich diet consisting of high levels of saturated fats (20) alters brain neurochemistry, particularly in the hypothalamus (21–23) where there exists populations of neurons that express several neurochemicals involved in controlling dietary fat intake (24–26). While the ingestion of a HFD can impact the levels of orexigenic and anorexigenic neuropeptides, the larger effect primarily occurs on the former (27, 28). Several orexigenic neuropeptides, with some of the main players to include enkephalin, galanin, orexin, and melanin-concentrating hormone, have been positively linked to HFD intake that increases expression and peptide levels in the brain. The injection of these neuropeptides or analogs of these neuropeptides directly into the hypothalamus can in turn stimulate excessive HFD intake (20, 29–33). The circuitry maintaining these peptide neurons are complex, with hormones released from the gut, such as ghrelin and cholecystokinin (34, 35), to directly impact hypothalamic peptide neuron excitation and inhibition, respectively, and stimulate or inhibit feeding behavior (**Figure 1**).

These early neuropeptide studies expanded to include local hypothalamic circuitry involved in the control of neuropeptide release. Two of the main classical inhibitory and excitatory neurotransmitters, γ-Aminobutyric acid (GABA) and glutamate, have been examined and have been shown to contribute to dietary-fat sensitive neuropeptide function. Glutamatergic innervation of the hypothalamus has been closely linked to ingestive behavior, with a glutamate analog injected into the hypothalamus or the surrounding intracerebroventricular region to increase food intake (36). To date, glutamatergic neuronal contacts with peptide neurons include neuropeptide Y (37), leptin (38), melanin-concentrating hormone (39), and orexin (40). Glutamatergic neurons that may be involved in stimulating the release of peptides from hypothalamic neurons during hunger to initiate food intake can also be depressed during short-term HFD intake and may act as a control mechanism to both initiate feeding and prevent over-ingestion of dietary fat. (41, 42). Linehan et al, found that excitatory signaling on orexin neurons could be depressed during short-term HFD intake but had reduced sensitivity to this signaling during long-term HFD regimes, suggesting extended glutamate signaling can exacerbate orexigenic neuropeptide signaling that leads to increased dietary fat intake (42). Long-term HFD exposure, in female mice in particular, can increase the number of glutamatergic neurons in the hypothalamus (43) and may over time increase neuronal signaling of peptide neurons to stimulate behavioral changes. Likewise, GABA agonists can exert similar effects as glutamate signaling in stimulating feeding (44). Both glutamatergic and GABAergic signaling in the brain can be impacted by overactivity and cellular stress. Prolonged exposure to a HFD can cause changes to the hypothalamic proteome involved in neuronal health and viability, and suggests these effects can lead to increased cellular stress, mitochondrial dysfunction, and altered synaptic plasticity (45), factors that impact glutamatergic and GABAergic signaling in the brain. These HFD-induced brain neurochemistry changes in adults leading to behavioral and physiological changes could potentially impact reproduction and offspring development.

**Figure 2:**
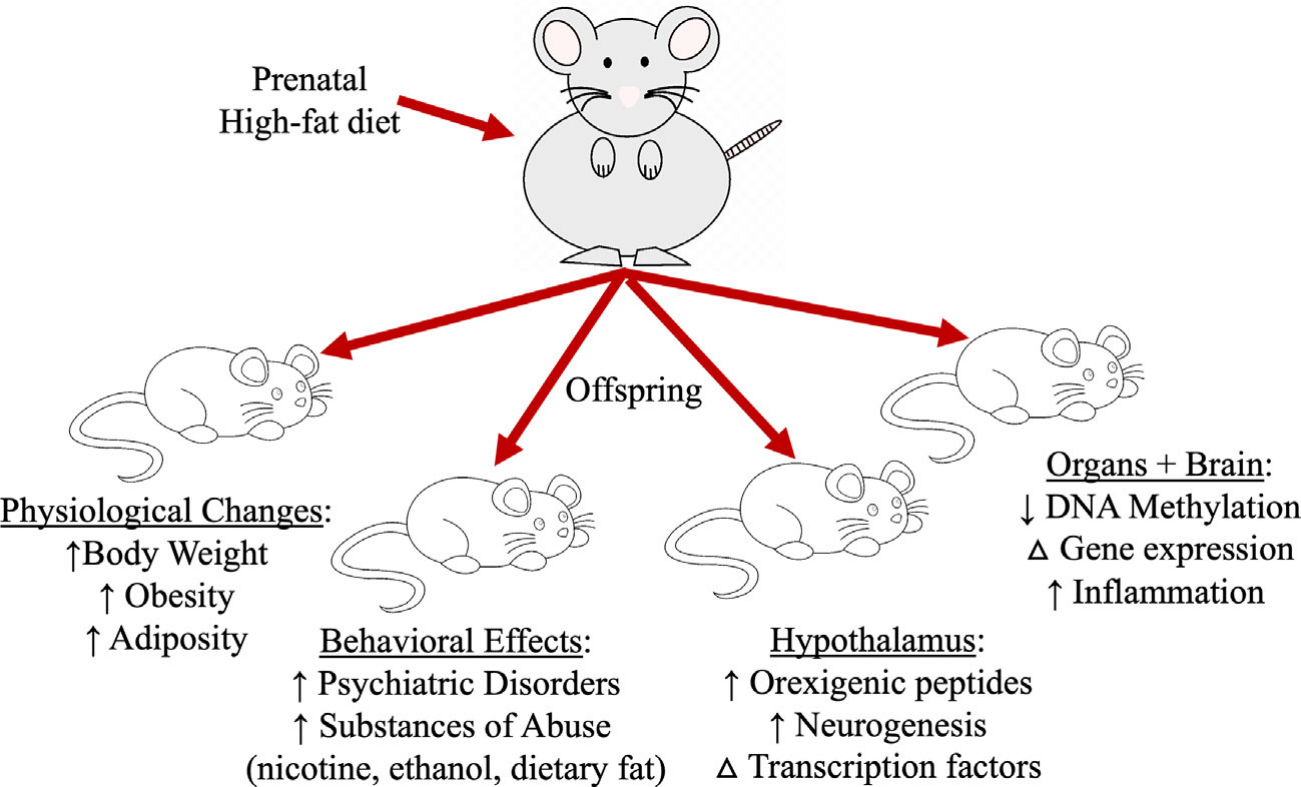
Prenatal high-fat diet exposure impacts several aspects of offspring physiology and behavior that leads to excessive dietary fat intake and increasing the risk for obesity.

The extensive documented effects of a HFD in adult animals on neuropeptides and neuronal connectivity is observed in offspring born from paternal or maternal obesity. The 1958 British birth cohort study show that both paternal and maternal obesity predisposes offspring to becoming obese, with maternal factors having a more pronounced phenotypic change in offspring compared to paternal obesity, and the combination of obesity in both parents have additive effects in offspring (17). In animal models, *in utero* exposure to a diet rich in fats with or without prior maternal obesity, predisposes offspring to have increased body weight (46–48), increases the risk of developing obesity (46), increases tendencies to consume other substances of abuse such as nicotine and ethanol (47, 49, 50), and is associated with an increase in the incidence of psychiatric disorders (**Figure 2**) (51–53). Ingestion of a HFD during pregnancy increases lipid accumulation in the placenta (54), while in offspring decreases the circulation of essential fatty acids (54), and increases the levels of triglycerides and adipocyte size (55, 56). Prenatal HFD paired with a postnatal HFD regime can cause several effects in offspring that is exerted on a macro and microscale level, including a reduction in the variation of DNA methylation in peripheral organs and changes the expression of genes involved in inflammation and RNA processing (48). The paternal inheritance of HFD-induced obesity paired with non-obese maternal pregnancy has similar physiological effects in offspring. Paternal obesity induces in male offspring increase in body weight, impairs glucose and insulin sensitivity and increases leptin levels while female offspring also has increased adiposity. Further breeding of these offspring reveals persistence of these physiological changes in F2 females but not males, suggesting HFD-induced obesity on the paternal side without maternal obesity contributes to female susceptibility to metabolic disorders (57, 58). Studies also show paternal gene imprinting of alleles related to body fat accumulation, Igf2 and Peg3, to be decreased in obesity-resistance mice compared to obesity-prone mice, suggesting a likelihood of paternal gene transmission in offspring resulting in diet-induced obesity (59). Although HFD-induced paternal obesity has been shown to effect physiological outcomes in offspring, the field has primarily focused on maternal contributions due to the large effects evoked in offspring brain neurochemistry.

In maternal models of HFD-intake, brain neurochemistry is changed such that offspring excessively consume dietary fat when presented with a high-fat diet choice (46, 60). This exposure during gestation increases in the hypothalamus of offspring the mRNA expression and peptide levels of the very same orexigenic neuropeptides that are altered during adult HFD intake (46). This increase is also accompanied by a pronounced rise in the neurogenesis of these peptide neurons (46, 61). While the relationship between dietary fat and neuropeptides in the hypothalamus is well established, the factors involved in causing neurochemical and neurogenesis changes in the brain of offspring are still under speculation, although many studies have provided some insight regarding specific pathways that may be involved. Prenatal HFD exposure can impact DNA methylation of several genes related to dietary fat intake (62, 63) as well as the levels and activity of several transcription factors that have been associated with brain development (64), such as transcriptional enhancer factor-1 (TEF-1), yes-associated protein-1 (YAP-1), and the family of peroxisome proliferator-activated receptors (PPARs), that in turn effect the expression of some of the orexigenic neuropeptides (65–68). The transcription factors TEF-1 and YAP-1 has been linked to prenatal HFD effects and plays a large role in organ formation and brain development during embryogenesis (69–72), and activation of these transcription factors have been found to control neuronal proliferation and differentiation (73–75). Both TEF-1 and YAP-1 were inactivated or decreased during *in utero* HFD exposure and may promote increased neurogenesis events (65). The PPAR family of transcription factors are fat sensitive and control lipid and carbohydrate metabolism and inflammation (76). While PPAR-δ is colocalized with hypothalamic ENK neurons, reduction of transcriptional regulator results in increased ENK expression and levels, suggesting a regulatory mechanism that is protective of HFD-exposure on neurons (66). A commonality of these factors is their relation to the immune system and the activation of inflammatory mediators. An abundance of research has directed the field toward inflammatory processes and inflammation, which are now thought to play a large role in both the physiological and central effects induced by the intake of a HFD and in obesity.

## Dietary Fat Intake Induces Global Systemic Inflammation

The ingestion of a HFD and the obese state produces an increase in systemic inflammation and has been reported to occur in almost every peripheral tissue and physiological system examined to date (77–80). This low-grade inflammation is characterized by an elevation of cytokines and chemokines (81, 82) that is atypical from the disease state and is accompanied by the activation of cellular inflammatory pathways (77, 83) and immune cells. This inflammation occurs whether HFD intake is acute or chronic and has been linked to diseases produced by metabolic syndrome. This inflammation has been linked to excessive levels of saturated fatty acids and omega-6 fatty acids in human western diets (84–86) and in HFD used in animal model studies (87–89).Central nervous system inflammation and activation of immune cells have also been linked to psychiatric disorders such as depression and bipolar disorder (90, 91), implicating the extensive effects HFD-induced inflammation has on the brain and behavior.

In the hypothalamus and in brain regions involved in the emotional component of ingestive behavior such as the amygdala and hippocampus (88, 89, 92, 93), there is an increase in many classical cytokines and activation of inflammatory pathways (94–96). These changes in inflammatory mediators can alter orexigenic neuropeptide levels in the hypothalamus (97–100), stimulate weight gain and increase HFD intake in animal models (101), while inhibition of hypothalamic inflammation with antibodies targeted for cytokines results in a reduction of food intake in obese animals (88, 96, 102). Neuroglial cells in the brain, including microglia, astrocytes, and oligodendrocytes, in conjunction with neurons, may contribute to this inflammation by releasing inflammatory mediators during exposure to excessive dietary fatty acids. Evidence suggests that the immediate inflammatory effect caused by a HFD in the brain can be due to several factors, including infiltration of peripheral macrophages (103), stimulation of resident microglia (94, 104), and direct astrocyte activation, which can increase inflammatory signaling and positively contribute to HFD intake and the onset of obesity (105, 106). The crosstalk between neuroglia, cytokine release, and dietary fat seem to directly impact neuronal signaling.

The exact pathway and the timing of the original inflammatory trigger across physiological systems and its subsequent effects in the brain is not known; is dietary fat itself the inflammatory trigger in the entire body or does the initial inflammatory trigger in the intestines where dietary fat is absorbed lead to additional inflammation across all physiological systems? For instance, long-term dietary fat intake can stimulate inflammation in the gut and alter intestinal microbiota leading to a leaky environment that is more susceptible to absorbing digested fats into the bloodstream (107, 108), with the increase in absorbed fatty acids to enter the brain and themselves induce central inflammatory effects. In contrast, reduction or alteration of gut microbiota results in a reduction of dietary fat intake (109, 110) and may prevent the central inflammatory effects by dietary fat. Based on this observation, it is possible that gut inflammation may precede brain inflammation (111) with the initial localized inflammation of the intestines producing central inflammatory effects. However, a single day of HFD intake itself can increase hypothalamic inflammation (77), suggesting that the increased load of absorbed fatty acids from the HFD itself may be directly causing the low-grade systemic inflammation in the body and brain, with longer term HFD ingestion to additionally contribute to gut inflammation and increase dietary fatty acid absorption that exacerbates the effects of dietary fat on the brain. This also suggests that the pathway of dietary fat intake and induction of inflammation conforms to a perpetual positive circuit and if continued over the course of reproductive age, can also lead to a generational effect.

## Maternal Inflammation Is Similar to Excessive Dietary Fat Intake

The impact of a HFD on inflammation is evidenced in maternal humans and in animal models that have widely established significant metabolic effects in offspring. In humans, increase of a marker of inflammation, C-reactive protein, induced by obesity has been correlated with increased incidence of obesity in offspring (112, 113) and suggests this inflammation may be involved in the prenatal programming of ingestive behavioral changes that leads to obesity. During pregnancy, several inflammatory mediators play a role in placental formation and in organogenesis events, and must maintain a delicate balance of immune function and immune suppression to ensure fetal survival (114–116). During pregnancy in obese humans, increased secretion of inflammatory mediators contributes to lowered blood flow into the placenta and potential dysfunction (113). It is possible that HFD-induction of a low-grade systemic inflammation (18) may disrupt the normal placental environment and impact the developing embryo. Prenatal exposure to a HFD and maternal obesity increases macrophage accumulation (117) and cytokine levels in the placenta (118, 119) that creates a more leaky environment to increase dietary fatty acids permeability through the placenta and into the embryo. Prenatal HFD also increases inflammation in several fetal organ systems that is found to be sustained in postnatal offspring, with this hyperinflammation to be further triggered during exposure to foreign pathogenic molecules (56, 119–121). The most notable physiological change in inflammatory profile is found in adipose tissue and closely mirrors the effect of a HFD in adult animals, revealing larger diameter adipocytes, upregulation of genes associated with adipogenesis, and increased association of these adipocytes with macrophages (56, 122, 123). One possibility for the increase in prenatal HFD associated inflammation may be due to the changes in maternal gut microbiota that result in increased intestinal inflammation and fatty acid absorption. The excess fatty acids and inflammatory mediators in the circulatory system of the mother in conjunction with the change in immune cell recruitment can allow these biological agents to enter the placenta and the fetus (119, 124, 125). These studies further show these microbiota changes in the mother to alter offspring microbiota and inflammatory profiles that persist postnatally and into adulthood (126–128). The combination of a HFD increasing inflammation in the mother and in the embryo may alter the course of brain development during gestation.

Prenatal HFD exposure increases in the hypothalamus of offspring the expression of cytokines and chemokines (104, 129, 130), compromises the blood brain barrier (131), the number of astrocytes (132), macrophage infiltration into the brain (103), activation of microglia (133), and immunoglobulin levels in microglia (94). These effects of a HFD in offspring on inflammation, immune cells, and inflammatory mediators can last well into adulthood (134–136) and has been linked to a number of disorders and diseases (137). This interplay between the immune system and HFD induced changes in fetal development is further solidified with prenatal inflammation challenges. Exposure to inflammatory mediators or challenges to the immune system during pregnancy results in increased body weight, increased risk of the development of obesity, and increased ingestive behavior in offspring (138, 139). In contrast, treatment during pregnancy with a natural anti-inflammatory compound, resveratrol, prevented brain changes and physiological changes associated with prenatal high-fat diet exposure (140, 141). The consequences of this heightened immune response in fetal brain may be rewiring of neuronal architecture that leads to postnatal behavioral and physiological consequences in offspring. These aspects of a HFD on maternal immune function, placental formation, and the fetus itself suggest that inflammatory mediators and immune cells play a unique and diverse role during pregnancy and embryogenesis.

## Modulation and Development of Hypothalamic Peptide Neurons by Chemokines

A unique class of inflammatory mediators that have recently been shown to play a role in the HFD effects on brain neurochemistry in offspring are the chemokines. Chemokines are a large class of small *chemo*tactic cyto*kines* that induce chemotactic activity in the immune system and has additional non-immunogenic function in the brain (142, 143). Generally, chemokines have been shown to regulate the development, migration and function of cells in several peripheral organs in addition to the brain, with genetic deletion of specific chemokines, such as C-C motif ligand 2 (CCL2) or C-X-C motif chemokine 12 (CXCL12), to modify embryonic development or in certain cases be lethal (144–146). For example, conditional genetic knockout of CXCL12 or its receptor CXCR4 in organ systems has been shown to result in defective blood vessel development in kidneys (147) and thicken alveolar tissue reducing oxygen exchange in lungs of mice (148). In an early 1990’s study conducted by Plata-Salaman CR, et al, they infused several chemokines into the intracerebroventricular zone in rats, measured feeding outcomes, and found that several chemokines reduced acute food intake, providing the first evidence showing the involvement of chemokines in the neuronal regulation of feeding (149). Since then, several chemokines have been linked to metabolism and brain neurochemicals, and given the nature of chemokines, may also be involved in the neurogenesis effects of a HFD on hypothalamic peptide neurons. The chemokine CXCL12 has been shown to be involved in neurogenesis events by regulating neuronal migration and proliferation in several brain regions (150–153) and controlling neuropositioning of newly-born neurons (154). Knockdown of CXCL12 causes impaired proliferation, migration, and differentiation of neurons (145, 155) suggesting modifications to this system can effect peptide neuron formation during pregnancy.

**Figure 3:**
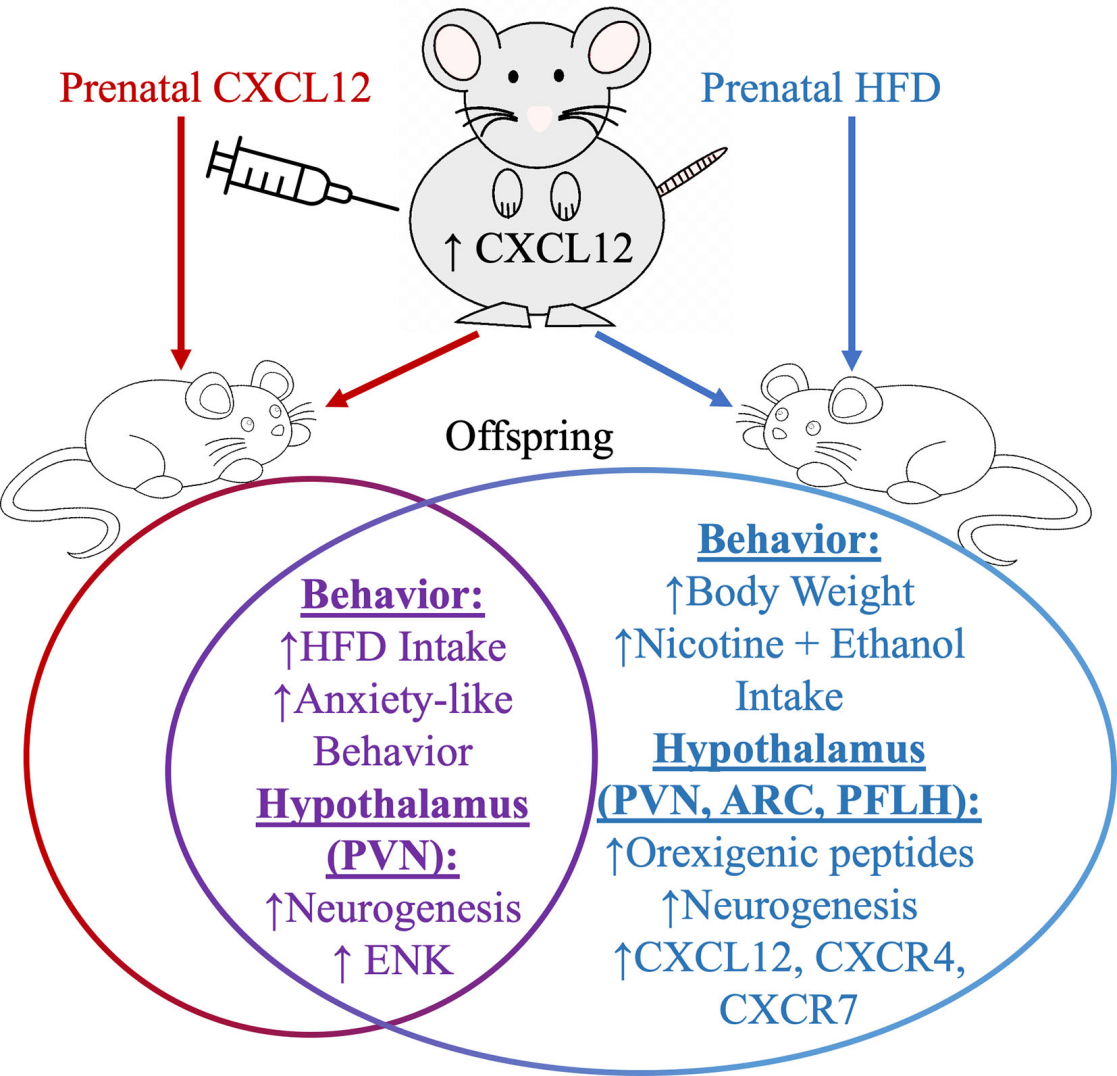
Prenatal high-fat diet ingestion can increase circulating levels of CXCL12 in pregnant rats and also induces in offspring several changes in both behavior and in orexigenic peptide neurons in the arcuate nucleus (ARC), perifornical lateral hypothalamus (PFLH), and in the paraventricular nucleus of the hypothalamus (PVN). Prenatal HFD and prenatal CXCL12 overlap in their effects in offspring on increasing ENK levels in the PVN, and in increasing high-fat diet intake and anxiety-like behaviors.

In adult rat models of HFD intake, the chemokine CXCL12 is increased in circulation and the brain while its receptors, C-X-C chemokine receptor 4 (CXCR4) and C-X-C chemokine receptor 7 (CXCR7), are increased in the hypothalamus and third ventricular injection of CXCL12 into the brain stimulates the expression of ENK in the paraventricular nucleus of the hypothalamus (87). Likewise, prenatal HFD exposure during the window of hypothalamus development significantly elevates maternal circulating levels of CXCL12 and also in offspring stimulates ENK expression in conjunction with CXCL12 and its receptors, CXCR4 and CXCR7, in the paraventricular nucleus of the hypothalamus (**Figure 3**). Maternal injection of CXCL12 during this same hypothalamic developmental time period in pregnancy to achieve chemokine levels similar to a prenatal HFD paradigm can increase the genesis of ENK neurons in the paraventricular nucleus of the hypothalamus in offspring (156) and produce similar effects in increasing anxiolytic and ingestive behavior in offspring. These studies suggest that one pathway of HFD in its physiological and behavioral effects may be mediated through the CXCL12 chemokine pathway, with this chemokine to directly effect ENK in the paraventricular nucleus of the hypothalamus.

Another chemokine that is transformed by and may be involved in the physiological and behavioral effects of a HFD is the chemokine, CCL2, also known as monocyte-chemoattractant protein-1 (MCP-1). This chemokine is known to be highly expressed in the obese state and is closely associated with the intake of a HFD (157), with genetic deletion or pharmacological blockade of its receptor, C-C chemokine receptor 2 (CCR2), to reverse or eliminate HFD-induced effect (158). This chemokine has much broader range in the brain due to its diverse co-expression with neuropeptides in several brain areas, including the hypothalamus and thalamus (159–161), and in developmental studies with other substances of abuse reveal close association with migratory radial glial cells (161). If the CCR2 receptor is centrally blocked by an antagonist, weight-loss induction by triggering foreign molecule inflammation through lipopolysaccharide injection is blocked (162), suggesting reduced brain activity of the CCR2 receptor may contribute to increased dietary fat intake and subsequent weight gain. In examining hypothalamic neurons extracted from embryos exposed to low-fat diet conditions, stimulation of hypothalamic neurons by CCL2 increases migration of hypothalamic neurons and the expression of the orexigenic peptides, enkephalin and galanin (159). In contrast, prenatal HFD exposure inhibited CCL2’s ability to produce these same effects (163), suggesting this diet severely disrupts the normal functioning of the CCL2/CCR2 signaling pathway in offspring brain to reduce over-ingestion of dietary fat. Other chemokines that have been implicated in the neuronal effects of a HFD include C-X3-C motif ligand 1 (CX3CL1) and C-C motif ligand 5 (CCL5) and their receptors (164, 165). The CX3CL1 is one of the first chemokines to be expressed during early hypothalamic inflammation (164), has been shown to be a marker for developing metabolic syndrome (166), its presence associated with increased propensity to develop obesity and increased recruitment of white blood cells into adipose tissue (167), and found to be highly expressed in the hypothalamus in HFD-induced obesity in mice (164). Reduction of CX3CL1 expression in the hypothalamus of mice provide mild reduction in brain inflammation and glucose tolerance, but not adiposity measures and body mass, suggesting this chemokine has a specific role in controlling HFD-induced obesity-related glucose metabolism (164). The chemokine CCL5 has been found to be co-expressed in the hypothalamus with insulin receptors and found to also be involved in insulin resistance derived from HFD-induced obesity (168). Intracerebroventricular injection of CCL5 stimulated the mRNA expression of orexin and melanin-concentrating hormone in the hypothalamus (169), suggesting this chemokine is another target of HFD effects that explicitly functions to activate these two fat-associated peptide neurons. A recent study by Fioravante et al, revealed that a chemokine decoy receptor, Ackr2, to be involved in the physiological and neurological effects of a HFD. This receptor is found in the hypothalamus and co-expressed in NPY and POMC neurons. After the intake of an acute HFD regimen, mice that were obesity-prone compared to obesity-resistant had much lower levels of the Akr2 receptor in the hypothalamus (170), suggesting that the increased presence of this decoy chemokine receptor reduces chemokine inflammation that occurs with HFD intake. With over 50 chemokines to date and a few studies showing individual chemokines to be involved in specific aspects of HFD-intake and obesity, future studies are needed to further clarify the role of individual chemokines in mediating HFD effects on brain development and ingestive behavior.

## Future Directions

While the extensive research in the field of ingestive behavior has uncovered a large body of knowledge that details how a HFD causes changes in the developing body and the brain, many questions still need to be addressed. While research has primarily focused on the maternal effects of HFD ingestion due to the larger effect on offspring, evidence shows that the paternal side can additively contribute to offspring phenotype and singularly effect female offspring more than male offspring. This suggests that future studies should focus on male contributions in the transmission of HFD-induced obesity proneness to offspring. There is a lack of brain studies in particular and examining the paternal effects on offspring brain neurochemistry and should be explored. While the extensive inflammatory effects induced by a HFD on the entire body is also well-documented, there are many players involved and, particularly with chemokines, shown to have specific function in mediating certain aspects of a HFD on development, cellular and neuronal profiles, and on behavior. It is therefore imperative to continue individualized studies on each inflammatory mediator to explore the function and role that each chemokine may play during HFD intake, particularly during the developmental period. Lastly, the penetration of maternal immune cells, inflammatory mediators, and fatty acids into the embryos during development is not known. Exploration of this topic could elucidate whether it is maternal HFD itself or maternal inflammation that is directly impacting developmental outcomes.

## Conclusions

Ingestion of a HFD and prenatal exposure to this diet has similar effects on activating inflammatory pathways in adult, embryonic, and postnatal animal models. The system-wide effects of dietary fat and the abundance of immune factors and ubiquitous expression of inflammatory mediators in peripheral organ systems and in the cells of the central nervous system suggest several regulatory pathways are involved in distinct aspects of ingestive behavior. While a few studies show positive results with the use of anti-inflammatory compounds to prevent prenatal changes in offspring, research on the long-term ramifications in offspring of non-selectively decreasing inflammation during pregnancy still needs to be performed. Similar to the individual chemokine studies such as with CXCL12, future studies focusing on relating each inflammatory mediator with the specific neuronal outcome and behavior may reveal the degree and the type of anti-inflammatory supplements to safely ingest during pregnancy as a preventative measure.

## Author Contributions

The author confirms being the sole contributor of this work and has approved it for publication.

## Conflict of Interest

The author declares that the research was conducted in the absence of any commercial or financial relationships that could be construed as a potential conflict of interest.
